# Recent Advances in Dynamic Kinetic Resolution by Chiral Bifunctional (Thio)urea- and Squaramide-Based Organocatalysts

**DOI:** 10.3390/molecules21101327

**Published:** 2016-10-14

**Authors:** Pan Li, Xinquan Hu, Xiu-Qin Dong, Xumu Zhang

**Affiliations:** 1 College of Chemical Engineering, Zhejiang University of Technology, Hangzhou 310014, Zhejiang, China; lpan760615496@163.com (P.L.); xinquan@zjut.edu.cn (X.H.); 2 College of Chemistry and Molecular Sciences, Wuhan University, Wuhan 430072, Hubei, China; 3 Department of Chemistry, South University of Science and Technology of China, Shenzhen 518000, Guangdong, China

**Keywords:** dynamic kinetic resolution, (thio)urea, squaramide

## Abstract

The organocatalysis-based dynamic kinetic resolution (DKR) process has proved to be a powerful strategy for the construction of chiral compounds. In this feature review, we summarized recent progress on the DKR process, which was promoted by chiral bifunctional (thio)urea and squaramide catalysis via hydrogen-bonding interactions between substrates and catalysts. A wide range of asymmetric reactions involving DKR, such as asymmetric alcoholysis of azlactones, asymmetric Michael–Michael cascade reaction, and enantioselective selenocyclization, are reviewed and demonstrate the efficiency of this strategy. The (thio)urea and squaramide catalysts with dual activation would be efficient for more unmet challenges in dynamic kinetic resolution.

## 1. Introduction

**Scheme 1:**
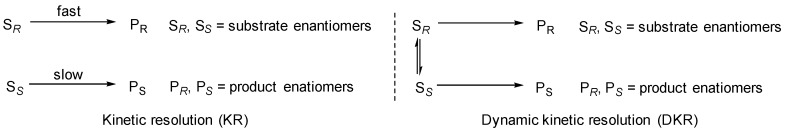
Kinetic resolution (KR) and dynamic kinetic resolution (DKR).

The synthesis of optically pure compounds is increasingly in demand in the pharmaceutical, fine chemicals, and agriculture industries [1,2,3,4,5,6,7,8]. Resolution of racemates is one of the most important industrial approaches to obtain enantiomerically pure compounds [9,10]. Kinetic resolution (KR) is a process in which one of the enantiomers of a racemic mixture is transformed to the corresponding product faster than the other one (Scheme 1) [11,12,13]. If the KR process is efficient, one of the enantiomers of the racemic mixture is completely transformed to the desired product while the other remains unchanged. Therefore, the critical drawback of KR process is the maximum theoretical yield of 50%. The dynamic kinetic resolution (DKR) process is an attractive strategy without this limitation, which efficiently combines the process of KR and the racemization of the slowly reacting enantiomer in a one-pot system with 100% theoretical yield (Scheme 1) [14,15,16,17,18,19,20]. This powerful strategy has been widely applied to a great many asymmetric catalytic reactions for the access of chiral compounds [21,22,23,24,25,26,27,28,29].

**Figure 1:**
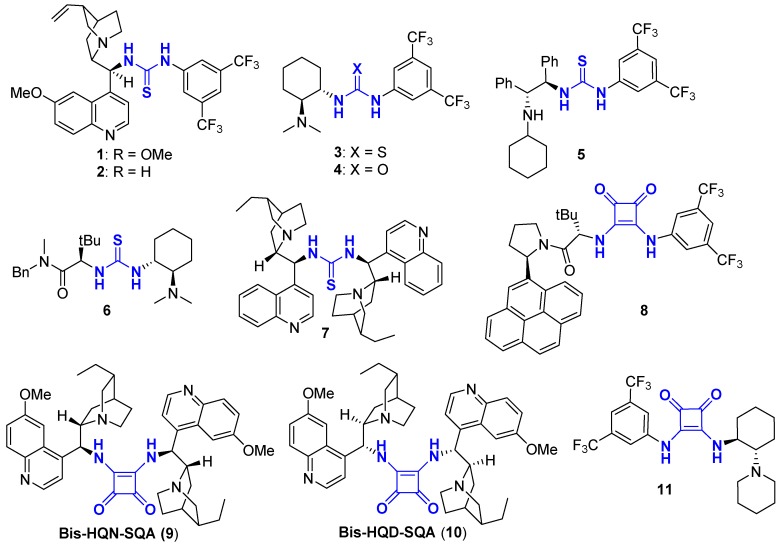
Selected reported representative (thio)urea and squaramide catalysts.

Asymmetric organocatalysis has been made significant advances in the last decades [30,31,32], and numerous asymmetric transformations were broadly applied to construct natural products [33] and industrial products [34]. Until now, various activation strategies have been developed, such as noncovalent catalysis via hydrogen-bonding [35], Brønsted base [36,37], Brønsted acid [38,39], phase transfer [40], and covalent catalysis via Lewis base [41]. Metal catalytic DKR has dominated in this research field during the end of the last century [42], and organocatalytic asymmetric reactions have reached maturity in recent years with impressive progress [43,44,45,46,47,48,49,50,51,52]. Among these chiral organocatalysts, chiral bifunctional (thio)ureas and squaramides catalysts have been intensively investigated to promote asymmetric reactions via dual hydrogen-bonding interactions which worked along with a Lewis acid functional group to achieve the activation of both the nucleophile and the electrophile [53]. Since the early research of primary amine–thiourea catalyzed the process of DKR in 2006 [54,55,56], DKR catalyzed by (thio)urea and squaramide has achieved tremendous advances [57,58,59,60,61,62,63,64,65,66]. The goal of the present review is to cover the recent developments concerning chiral (thio)ureas and squaramides (Figure 1) catalytic reactions through DKR. This review is subdivided into two sections according to the types of organocatalysts employed in these asymmetric reactions involving the process of DKR.

## 2. (Thio)urea Organocatalyst for DKR

### 2.1. Thio-Michael–Michael Cascade Reaction

**Scheme 2:**
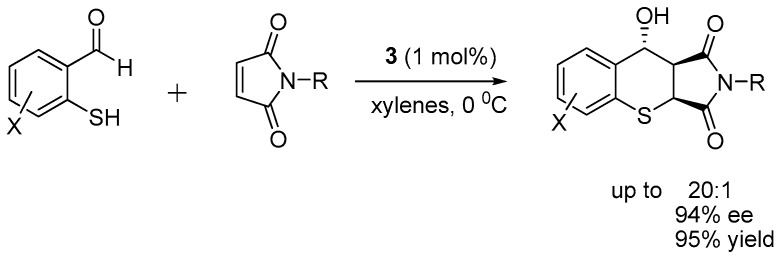
Asymmetric thio-Michael–aldol cascade reaction of 2-mercaptobenzaldehydes with maleimides catalyzed by **3**.

Asymmetric (thio)urea catalyzed Michael–Michael cascade reactions are one of the most powerful strategies for the efficient construction of chiral complex molecules with multiple bonds’ formation and multistereogenic centers’ creation [67,68]. In 2007, Wang and his coworker explored the novel thio-Michael–aldol reaction, which employed 2-mercaptobenzaldehydes and maleimides as template substrates and ligand **3** as catalyst. With the standard conditions, excellent yield and good stereoselectivity was achieved (90% yield, 84% ee, 10:1 dr). Further examination of the scope proved that this new methodology was a general approach to the preparation of a range of substituted thiochromanes (Scheme 2) [67]. 

**Scheme 3:**
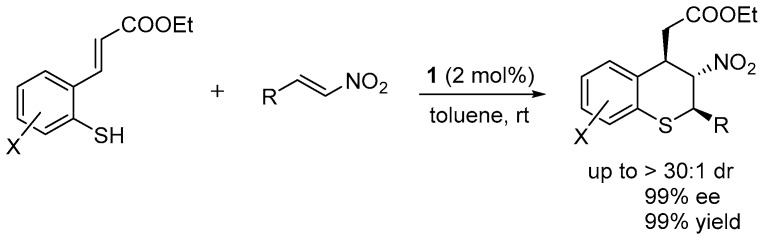
Asymmetric thio-Michael–Michel cascade reaction of *trans*-3-(2-mercapto-aryl)-2-propenoic acid ethyl esters with nitroolefins catalyzed by **1**.

In 2008, Wang and coworkers developed a highly stereoselective thio-Michael-Michael cascade reaction of *trans*-3-(2-mercaptophenyl)-2-propenoic acid ethyl ester and *trans*-β-nitrostyrene catalyzed by a cinchona alkaloid-derived thiourea (**1**), which afforded chiral thiochromanes with three new stereogenic centers through one-pot access in excellent efficiency and stereoselectivity (Scheme 3, up to dr > 30:1, 99% ee, 99% yield) [68]. The generality of this cascade reaction was very wide, and a series of nitroalkenes can proceed well. In addition, this cascade reaction involved the DKR process, which has made great contributions to this transformation.

**Scheme 4:**
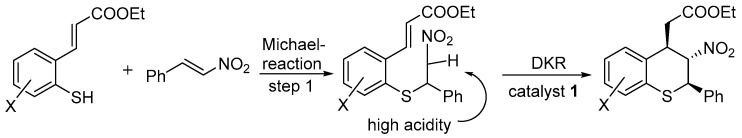
Proposed pathway for asymmetric thio-Michael–Michael cascade reaction.

The initially formed Michael addition product, through reversible thia-Michael addition step 1 with strong acidity, went through deprotonation in the presence of bifunctional cinchona alkaloid-derived thiourea **1**, and then underwent a DKR process, which is mediated by the chiral bifunctional amine thiourea **1** and followed with retro-Michael–Michael–Michael process, providing chiral thiochromane product (Scheme 4). Moreover, this hypothesis was confirmed by the treatment of racemic first Michael adduct with catalyst **1** under the standard reaction conditions, affording similar reaction results.

**Scheme 5:**
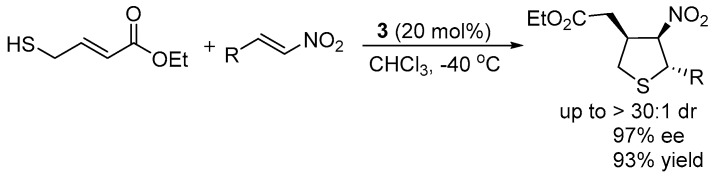
Asymmetric thio-Michael–Michael cascade reaction of *trans*-ethyl 4-mercapto-2-butenoate with nitroolefins catalyzed by amine–thiourea **3**.

In 2011, Wang and coworkers established another asymmetric thio-Michael–Michael cascade reaction of *trans*-ethyl 4-mercapto-2-butenoate and *trans*-β-nitrostyrene catalyzed by Takemoto’s bifunctional amine–thiourea catalyst (**3**), and the functionalized chiral trisubstituted tetrahydrothiophene products bearing three stereogenic centers through sequential C–S and C–C bond formation with high enantio- and diastereoselectivity (Scheme 5, up to >30:1 dr, 97% ee, 93% yield) [69]. Various aromatic nitroolefins performed smoothly in this transformation. The heteroaromatic and less reactive alkyl *trans*-β-nitroolefins were also well tolerated. In addition, an unprecedented activation mode of cooperative direct stereocontrol and similar DKR process was identified. 

**Scheme 6:**
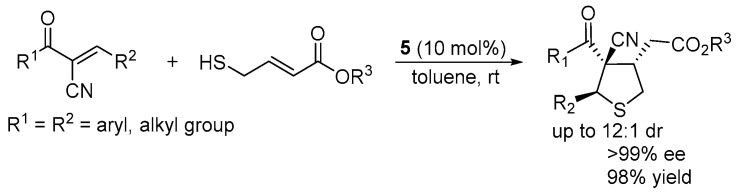
Asymmetric thio-Michael–Michael cascade reaction of *trans*-α-cyano-α,β-unsaturated ketones with *trans*-4-mercapto-2-butenoates catalyzed by amino thiourea **5**.

In 2013, Lattanzi and coworkers successfully disclosed an efficient cascade double Michael reaction of *trans*-α-cyano-α,β-unsaturated ketone with *trans*-4-mercapto-2-butenoate to construct chiral trisubstituted tetrahydrothiophenes catalyzed by amino thiourea **5** with excellent results (Scheme 6, up to 12:1 dr, >99% ee, 98% yield) [70]. The tetrahydrothiophene products contained three stereocenters, and one challenging all-carbon quaternary stereocenter was successfully created.

**Scheme 7:**
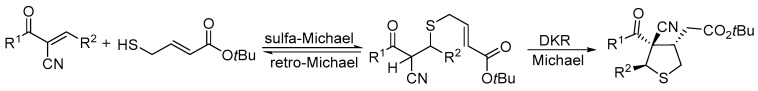
The cascade pathway involving DKR process.

In addition, the control experiment confirmed that the DKR process was involved in this asymmetric cascade reaction (Scheme 7). The racemic mixture of diastereoisomers of the first sulfa-Michael product was treated under the optimized reaction conditions in the presence of catalyst **5**, and the result was the same with the *trans*-α-cyano-α,β-unsaturated ketone reacting with *trans*-4-mercapto-2-butenoate directly.

### 2.2. Asymmetric Ring Opening of Azlactones

**Scheme 8:**
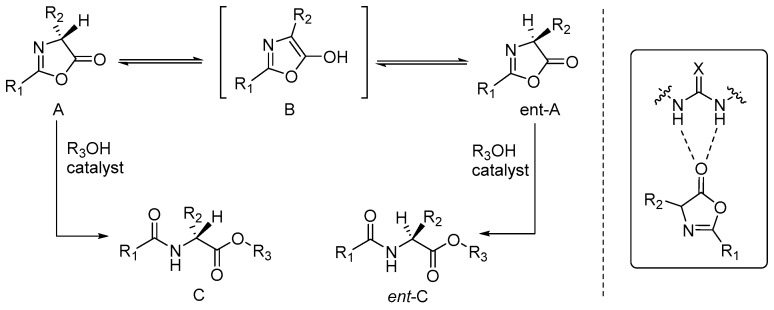
Basis of the DKR of azlactones.

Chiral α-amino acids have been widely used for the construction of pharmaceuticals, nature products, ligands, and organocatalysts [71,72,73,74]. The alcoholytic dynamic kinetic resolution of azlactones has been regarded as an attractive and important way to generate the enantiomerically enriched α-amino acid derivatives (Scheme 8). The azlactones are readily prepared by the Erlenmeyer azlactone synthesis or from amino acids by N-acylation followed by cyclization–dehydration in the presence of condensation reagent [75].

**Scheme 9:**
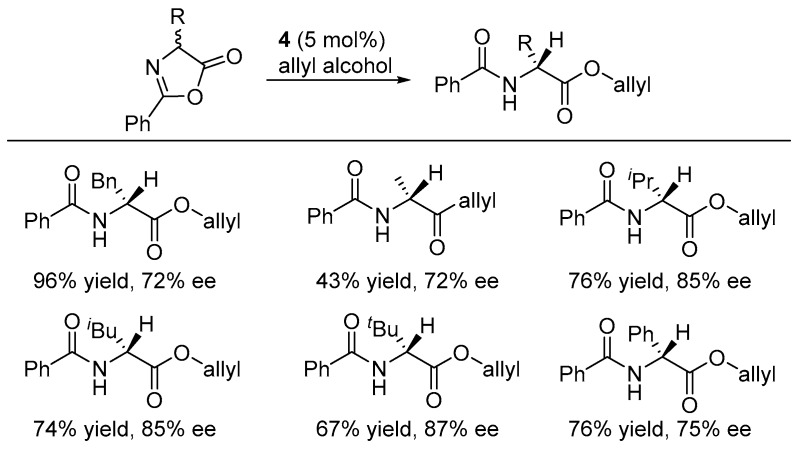
Asymmetric ring opening of azlactones with allyl alcohol by catalyst **4**.

In 2005, Berkessel successfully developed asymmetric ring opening of azlactones with alcohol via the DKR process catalyzed by bifunctional amine urea catalyst **4** (Scheme 9) [76], which demonstrated the effective hydrogen-bonding activation of the (thio)urea moiety with the azlactone substrates. The NMR-spectroscopic studies also indicated that the catalyst activates the substrate azlactone by hydrogen bonding of the (thio)urea moiety directing to the carbonyl oxygen atom. The alcoholysis and ring opening of these substrate azlactones—derived from the aliphatic α-amino acids phenylalanine, alanine, valine, leucine and *tert*-leucine, and the aromatic α-amino acid phenylglycine—proceeded efficiently with good enantioselectivities catalyzed by amine urea **4** (72%–87% ee).

**Scheme 10:**
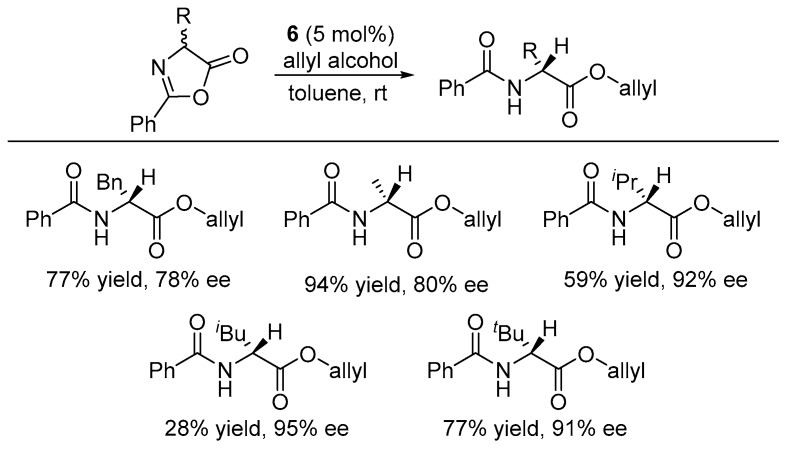
Asymmetric ring opening of azlactones with allyl alcohol by catalyst **6**.

**Scheme 11:**
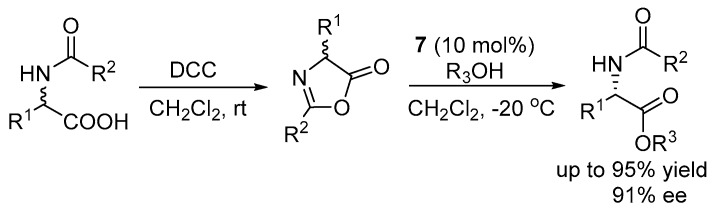
One-pot procedure for the DKR reactions.

Based on their previous studies, Berkessel and coworkers envisioned that the reactivity and enantioselectivity of this transformation could be greatly improved by reasonable modified thiourea catalyst [77]. The initial catalyst-screening results of Takemoto-type bifunctional organocatalysts demonstrated that increasing the steric hindrance of the additional chiral center may contribute to enhancement of enantioselectivity. Therefore, they synthesized a series of the *tert*-leucine amide-derived catalysts, and catalyst **6** was identified to be the best one. Various azlactones derived both from natural and non-natural α-amino acids were investigated to examine the generality of substrates in the presence of catalyst **6**, and the enantioselectivity was up to 95% (Scheme 10). They successfully realized clean *stereoinversion* of natural and non-natural α-amino acids through this organocatalytic DKR.

(Thio)urea-based organocatalysts, to some extent, existed hydrogen-bonded aggregates, which led to realization that the reactivity and enantioselectivity were greatly dependent on concentration and temperature of reactions [78,79,80]. The enantioselectivity always dramatically decreased when the concentration was increased, and this was not conducive to their practical application. In 2012, Song and coworkers reported that C2-symmetric bis-cinchona-alkaloid-based thiourea **7** was applied to catalyze the DKR of racemic azlactones available from N-protected racemic amino acids in one pot, affording various chiral non-natural α-amino esters (Scheme 11, up to 95% yield, 91% ee) [81]. The steric bulkiness of the two alkaloid moieties of catalyst **7** can prevent their self-aggregation and exhibited concentration-independent enantioselectivity in this transformation. Moreover, the experimental and NMR-spectroscopic studies and single crystal X-ray analysis confirmed that these kinds of bifunctional organocatalysts do not establish hydrogen-bonded self-aggregates in either solution or solid state.

### 2.3. Atropo-Enantioselective Transesterification

Asymmetric synthesis of axially chiral biaryl compounds emerged as a challenging and attractive research area, where they were found to have broad applications for constructing various natural products, drugs, bioactive molecules, and chiral ligands [82,83,84,85,86]. There are numerous excellent synthetic methodologies to efficiently build chiral biaryl skeletons, such as chiral auxiliaries, asymmetric transformations, asymmetric oxidation homo couplings, and asymmetric Suzuki–Miyaura couplings [87,88,89,90,91,92]. DKR of configurationally labile biaryl lactones was a powerful and outstanding transformation to produce such chiral compounds and continues to receive increasing attention now [93,94,95,96,97,98]. Owing to great versatile application and highly effective activation mode of bifunctional chiral thiourea catalyst, the combination of DKR and organocatalysis is an attractive way to build chiral biaryl compounds.

**Scheme 12:**
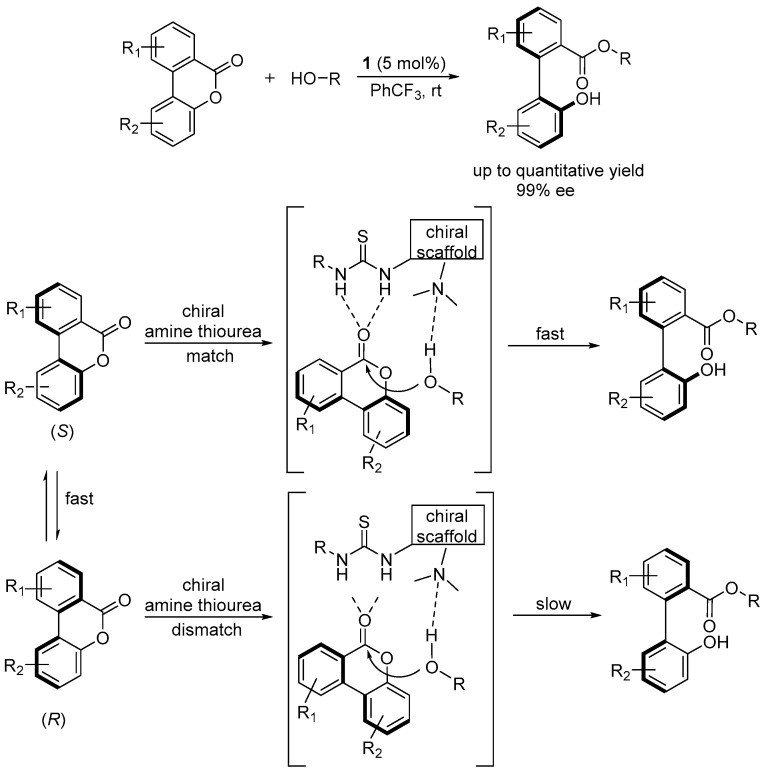
DKR of biaryl lactones via bifunctional amine thiourea (**1**)-catalyzed atropo-enantioselective transesterification.

Recently, Wang and coworkers realized highly atropo-enantioselective transesterification of biaryl lactones catalyzed by chiral bifunctional amine thiourea **1** provided enantioenriched axially chiral biaryl compounds with a wide substrate scope under mild reaction conditions (Scheme 12, up to quantitative yield, 99% ee) [99]. Additionally, this asymmetric transformation involved a highly enantioselective DKR process, which was owing to synergistic activation of the biaryl lactones and alcohols/phenols by the thiourea **1** and amine groups (Scheme 12).

## 3. Squaramide Organocatalyst for DKR

Asymmetric organocatalysis employing a hydrogen-bonding activation strategy has been well-established for the synthesis of enantioenriched compounds. Chiral squaramides have been identified as a type of powerful bifunctional hydrogen-bonding catalysts, and promoted numerous catalytic asymmetric transformations [42,43,44,45,46,47,48,49,50,51]. They were also effective for the asymmetric reactions involving the DKR process.

### 3.1. Enantioselective Selenofunctionalization Reactions

**Scheme 13:**
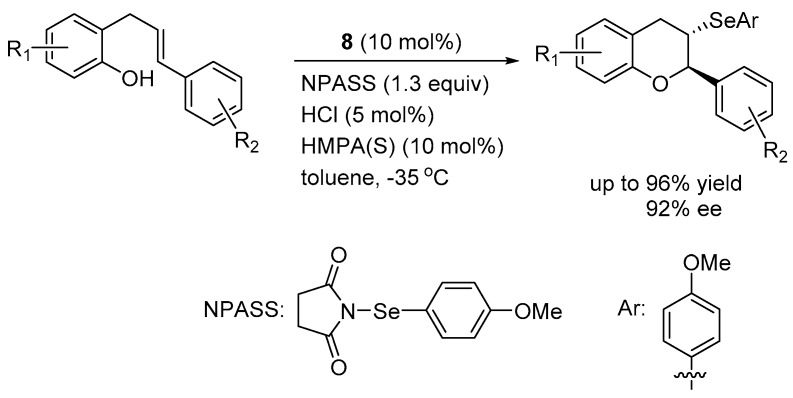
Asymmetric selenocyclization via DKR of seleniranium ions by squaramide catalyst **8**.

Asymmetric selenofunctionalization of alkenes was regarded as an attractive and challenging methodology to produce chiral selenide compounds in organic synthesis [100,101,102,103,104,105,106]. Due to the configurational instability of seleniranium ions, the rapid seleniranium racemization can contribute to promotion of the DKR process. In 2014, Jacobsen and coworkers successfully established highly enantioselective selenocylization reactions of *o*-allyl-substituted phenol via the DKR process of seleniranium ions by chiral squaramide catalyst **8** (Scheme 13, up to 96% yield, 92% ee) [107]. They made use of N-phenylselenyl succinimide (NPSS) as the selenium donor, and hydrogen chloride and tris-(dimethylamino)phosphorus sulfide (HMPA(S)) as cocatalysts. The substrates with different substituent patterns performed smoothly, affording cyclization products in high yield and enantioselectivity. However, the *ortho* substituent of the hydroxyl group substrate obtained poor enantioselectivity, which suggested that the possible interaction between the hydroxyl group and catalyst that may play an important role in the mechanism of enantioinduction, and such an interaction may be sensitive to the steric environment around the hydroxyl group.

**Scheme 14:**
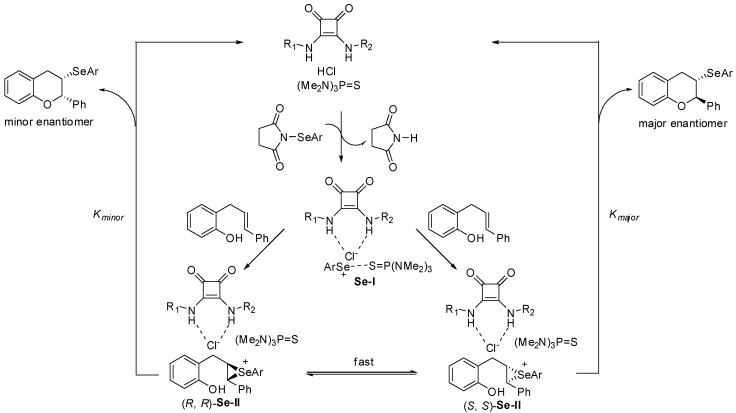
Proposed possible catalytic cycle.

According to the proposed activation strategy of this asymmetric selenocyclization, cooperative Lewis base and Brønsted acid activation of the electrophilic selenium reagent formed a reactive ion pair (**Se-I**), which may be associated with the squaramide catalyst **8**. This intermediate reacted with *o*-allyl-substituted phenol substrate and formed chiral seleniranium ions (*R*, *R*)-**Se-II** and (*S*, *S*)-**Se-II**. These two seleniranium ions equilibrated very rapidly, and subsequently went through cyclizations with different rates due to the association with chiral squaramide **8**, resulting in formation of products with excellent enantioselectivities (Scheme 14).

### 3.2. Asymmetric Ring Opening of Azlactones

**Scheme 15:**
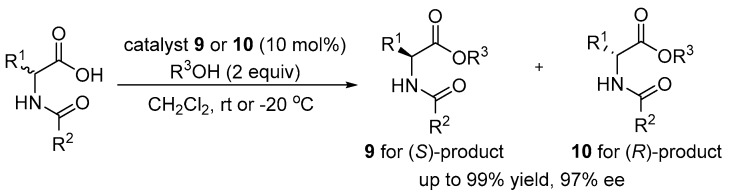
DKR of the racemic azlactones with alcohols by squaramide-based dimeric cinchona alkaloid catalysts.

Numerous thiourea-organocatalytic DKRs of racemic azlactones have been widely investigated, but few examples concerning DKR of racemic azlactones promoted by squaramide-organocatalysts were reported. Song and coworkers developed a novel catalytic DKR of ring-opening reactions of racemic azlactones with various alcohols by the bifunctional squaramide-based dimeric cinchona alkaloid catalysts **9** and **10** (Scheme 15) [108]. These catalysts displayed unprecedented catalytic activity, as well as enantioselectivity in the DKR reaction of a broad range of racemic azlactones (up to 99% yield, 97% ee). The recyclability of the catalysts was examined, and the enantioselectivity and activity were not decreased even after fifth run.

**Scheme 16:**
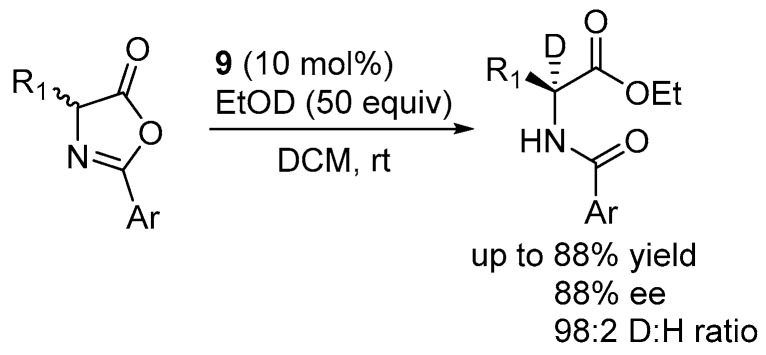
Catalytic DKR of various azlactones with ethanol-*d*.

Inspired by the aforementioned results, Song and coworkers further employed this protocol for the preparation of α-carbon deuterium-labeled α-amino acids with EtOD as a nucleophile as well as a deuterium source catalyzed by bifunctional squaramide-based dimeric cinchona alkaloid catalyst **9** (Scheme 16) [109]. Various α-deuterated amino esters were afforded with good enantioselectivities and yields (up to 88% yield, 88% ee). In addition, most of the *N*-protected α-deuterated amino ester products were obtained with a deuterium content greater than 95%, and their optical purity can be improved to >99% ee after a single recrystallization.

### 3.3. Cascade Sulfur-Michael–Michael Reaction

**Scheme 17:**
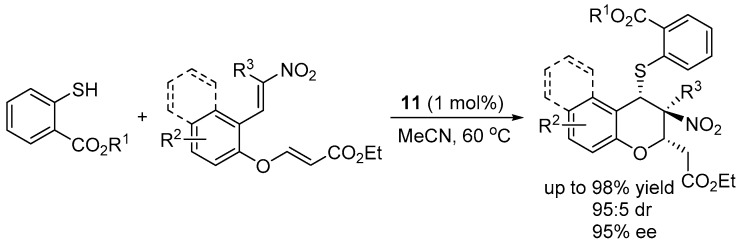
Squaramide-catalyzed asymmetric cascade sulfa-Michael/Michael addition of thiosalicylates to nitroalkene enoates.

Chroman is a kind of fundamental heterocyclic skeleton and widely found in natural products and medical molecules with great importance [110,111,112]. Organocatalytic cascade reactions were regarded as a straightforward and efficient method to construct chiral chroman framework [113]. In 2013, Du and coworkers developed an efficient asymmetric cascade sulfa-Michael/Michael addition of thiosalicylates with nitroalkene enoates to build chiral chromans catalyzed by a chiral bifunctional squaramide-tertiary amine catalyst **11** (Scheme 17, 98% yield, 95:5 dr, 95% ee) [114]. The highly functionalized chiral chroman products contained three contiguous stereocenters, including one quaternary center. Based on the results of control experiments, they proposed a reasonable reaction pathway of sulfa-Michael/retro-sulfa-Michael/sulfa-Michael/Michael reactions involving the DKR process.

## 4. Conclusions

Asymmetric organocatalysis is one of the most important research areas in organic synthesis and applicable in a broad variety of reaction types, which includes those reactions involving the DKR process. In this review, we have summarized recent significant developments on the catalytic asymmetric reactions via the DKR process promoted by the (thio)urea and squaramide organocatalysts. These reaction examples have confirmed that (thio)urea and squaramide organocatalysts bearing hydrogen-bonding donors as catalysts have played an important role in achieving excellent results from the transformations involving the DKR process. In addition, further exciting and significant discoveries of (thio)urea and squaramide organocatalytic DKR process and developments with this versatile type of bifunctional organocatalysis are to be expected in the near future with the advent of more systematic studies. 

## References

[1] Farina V., Reeves J.T., Senanayake C.H., Song J.J. (2006). Asymmetric synthesis of active pharmaceutical ingredients. Chem. Rev..

[2] Bornscheuer U.T., Huisman G.W., Kazlauskas R.J., Lutz S., Moore J.C., Robins K. (2012). Engineering the third wave of biocatalysis. Nature.

[3] Muñoz Solano D., Hoyos P., Hernáiz M.J., Alcántara A.R., Sánchez-Montero J.M. (2012). Industrial biotransformation in the synthesis of building blocks leading to enantiopure drugs. Bioresour. Technol..

[4] Sanchez S., Demain A.L. (2011). Enzymes and bioconversion of industrial, pharmaceutical, and biotechnological significance. Org. Process Res. Dev..

[5] Rajagopalan A., Kroutil W. (2011). Biocatalytic reaction: Selected highlights. Mater. Today.

[6] Nestl B.M., Nebel B.A., Hauer B. (2011). Recent progress in industrial biocatalysis. Curr. Opin. Chem. Biol..

[7] Jungbauer A. (2011). Improved products and processes through biochemical engineering science. Biotechnol. J..

[8] Tufvesson P., Fu W., Jensen J.S., Woodley J.M. (2010). Process consideration for the scale-up and implementation of biocatalysis. Food Bioprod. Process..

[9] Lorenz H., Seidel-Morgenstern A. (2014). Processes to Separate Enantiomers. Angew. Chem. Int. Ed..

[10] Pellissier H. (2011). Organocatalyzed dynamic kinetic resolution. Adv. Synth. Catal..

[11] Keith J.M., Larrow J.F., Jacobsen E.N. (2001). Practical Considerations in Kinetic Resolution Reactions. Adv. Synth. Catal..

[12] Vedejs E., Jure M. (2005). Efficiency in nonenzymatic kinetic resolution. Angew. Chem. Int. Ed..

[13] Robinson D.E.J.E., Bull S.D. (2003). Kinetic resolution strategies using non-enzymatic catalysts. Tetrahedron Asymmetry.

[14] El Gihani M.T., Williams J.M.J. (1999). Dynamic kinetic resolution. Curr. Opin. Chem. Biol..

[15] Kim M.-J., Ahn Y., Park J. (2002). Dynamic kinetic resolutions and asymmetric transformations by enzymes coupled with metal catalysis. Curr. Opin. Biotechnol..

[16] Pàmies O., Bäckvall J.-E. (2004). Chemoenzymatic dynamic kinetic resolution. Trends Biotechnol..

[17] Martín-Matute B., Bäckvall J.-E. (2007). Dynamic kinetic resolution catalyzed by enzymes and metals. Curr. Opin. Chem. Biol..

[18] Ahn Y., Ko S.-B., Kim M.-J., Park J. (2008). Racemization catalysts for the dynamic kinetic resolution of alcohols and amines. Coord. Chem. Rev..

[19] Lee J.H., Han K., Kim M.-J., Park J. (2010). Chemoenzymatic dynamic kinetic resolution of alcohols and amines. Eur. J. Org. Chem..

[20] Turner N. (2010). Deracemisation methods. Curr. Opin. Chem. Biol..

[21] Kamal A., Azhar M.A., Krishnaji T., Malik M.S., Azeeza S. (2008). Approaches based on enzyme mediated kinetic to dynamic kinetic resolution: A versatile route for chiral intermediates. Coord. Chem. Rev..

[22] Warner M.C., Casey C.P., Bäckvall J.E. (2011). Bifunctional Molecular Catalysis in Topics in Organometallic Chemistry.

[23] Pellissier H. (2011). Recent developments in dynamic kinetic resolution. Tetrahedron.

[24] Ahmed M., Kelly T., Ghanem A. (2012). Applications of enzymatic and non-enzymatic methods to access enantiomerically pure compounds using kinetic resolution and racemisation. Tetrahedron.

[25] Hoyos P., Pace V., Alcántara A.R. (2012). Dynamic kinetic resolution via hydrolase-metal combo catalysis in stereoselective synthesis of bioactive compounds. Adv. Synth. Catal..

[26] Warner M.C., Bäckvall J.-E. (2013). Mechanistic aspects on cyclopentadienylruthenium complexes in catalytic racemization of alcohols. Acc. Chem. Res..

[27] Denard C.A., Hartwig J.F., Zhao H. (2013). Multistep one-pot reactions combining biocatalysts and chemical catalysts for asymmetric synthesis. ACS Catal..

[28] Rachwalski M., Vermue N., Rutjes F.P.J.T. (2013). Recent advances in enzymatic and chemical deracemisation of racemic compounds. Chem. Soc. Rev..

[29] Ramon D.J., Yus M. (2006). In the arena of enantioselective synthesis, Titanium complexes wear the laurel wreath. Chem. Rev..

[30] List B. (2007). Introduction: Organocatalysis. Chem. Rev..

[31] Moyano A., Rios R. (2011). Asymmetric Organocatalytic Cyclization and Cycloaddition Reactions. Chem. Rev..

[32] Dalko P. (2013). Comprehensive Enantioselective Organocatalysis.

[33] Grondal C., Jeanty M., Enders D. (2010). Organocatalytic cascade reactions as a new tool in total synthesis. Nat. Chem..

[34] Howell G.P. (2012). Asymmetric and diastereoselective conjugate addition reaction: C–C bond formation at large scale. Org. Process Res. Dev..

[35] Pihko P.M. (2009). Hydrogen Bonding in Organic Synthesis.

[36] Doyle A.G., Jacobsen E.N. (2007). Small-molecule H-bond donors in asymmetric catalysis. Chem. Rev..

[37] Palomo C., Oiarbide M., López R. (2009). Asymmetric organocatalysis by chiral Bronsted bases: Implications and applications. Chem. Soc. Rev..

[38] Akiyama T. (2007). Stronger Brønsted acids. Chem. Rev..

[39] Terada M. (2010). Chiral phosphoric acids as versatile catalysts for enantioselective transformations. Synthesis.

[40] Maruoka K. (2008). Asymmetric Phase Transfer Catalysis.

[41] Paull D.H., Abraham C.J., Scerba M.T., Alden-Danforth E., Lectka T. (2008). Bifunctional asymmetric catalysis: Cooperative lewis acid/base systems. Acc. Chem. Res..

[42] Kim C., Park J., Kim M.J. (2012). Comprehensive Chirality.

[43] Takemoto Y. (2005). Recognition and activation by ureas and thioureas: Stereoselective reactions using ureas and thioureas as hydrogen-bonding donors. Org. Biomol. Chem..

[44] Connon S.J. (2006). Organocatalysis mediated by (Thio)urea derivatives. Chem. Eur. J..

[45] Storer R.I., Aciro C., Jones L.H. (2011). Squaramides: Physical properties, synthesis and applications. Chem. Soc. Rev..

[46] Han X., Zhou H.-B., Dong C. (2016). Applications of chiral squaramides: From asymmetric organocatalysis to biologically active compounds. Chem. Rec..

[47] Tian L., Hu X.-Q., Li Y.-H., Xu P.-F. (2013). Organocatalytic asymmetric multicomponent cascade reaction via 1,3-proton shift and [3+2] cycloaddition: An efficient strategy for synthesis of oxindole derivatives. Chem. Commun..

[48] Badiola E., Fiser B., Gómez-Bengoa E., Mielgo A., Olaizola I., Urruzuno I., García J.M., Odriozola J.M., Razkin J., Oiarbide M., Palomo C. (2014). Enantioselective construction of tetrasubstituted stereogenic carbons through Bronsted Base catalyzed Michael reaction: α′-hydroxy enones as key enoate equivalent. J. Am. Chem. Soc..

[49] Cao Y.-M., Shen F.-F., Zhang F.-T., Zhang J.-L., Wang R. (2014). Catalytic asymmetric 1,2-addition of α-isothiocyanato phosphonates: Snthesis of chiral β-hydroxy- or β-amino-substituted α-amino phosphonic acid derivatives. Angew. Chem. Int. Ed..

[50] Zhao B.-L., Du D.-M. (2015). Chiral squaramide-catalyzed Michael/alkylation cascade reaction for the asymmetric synthesis of nitro-spirocyclopropanes. Eur. J. Org. Chem..

[51] Sigman M.S., Jacobsen E.N. (1998). Schiff Base Catalysts for the Asymmetric Strecker Reaction Identified and Optimized from Parallel Synthetic Libraries. J. Am. Chem. Soc..

[52] Malerich J.P., Hagihara K., Rawal V.H. (2008). Chiral Squaramide Derivatives are Excellent Hydrogen Bond Donor Catalysts. J. Am. Chem. Soc..

[53] Huang H., Jacobsen E.N. (2006). Highly enantioselective direct conjugate addition of ketones to nitroalkenes promoted by a chiral primary amine-thiourea catalyst. J. Am. Chem. Soc..

[54] Chauhan P., Mahajan S., Kaya U., Hack D., Enders D. (2015). Bifunctional amine-squaramides: Powerful hydrogen-bonding organocatalysts for asymmetric domino/cascade reactions. Adv. Synth. Catal..

[55] Held F.E., Tsogoeva S.B. (2016). Asymmetric cycloaddition reactions catalyzed by bifunctional thiourea and squaramide organocatalysts: Recent advances. Catal. Sci. Technol..

[56] Tsakos M., Kokotos C.G. (2013). Primary and secondary amine-(thio)ureas and squaramides and their application in asymmetric organocatalysis. Tetrahedron.

[57] Wang W., Li H., Wang J., Zu L. (2006). Enantioselective organocatalytic tandem Michael-aldol reactions: One-pot synthesis of chiral thiochromenes. J. Am. Chem. Soc..

[58] Rios R., Sundén H., Ibrahem I., Zhao G.L., Eriksson L., Córdova A. (2006). Highly enantioselective synthesis of 2*H*-1-benzothiopyrans by a catalytic domino reaction. Tetrahedron Lett..

[59] Zu L.S., Wang J., Li H., Xie H.X., Jiang W., Wang W. (2007). Cascade Michael-aldol reactions promoted by hydrogen bonding mediated catalysis. J. Am. Chem. Soc..

[60] Li H., Wang J., E-Nunu T., Zu L., Jiang W., Wei S., Wang W. (2007). One-pot approach to chiral chromenes via enantioselective organocatalytic domino oxa-Michael-Aldol reaction. Chem. Commun..

[61] Wang X.-F., Hua Q.-L., Cheng Y., An X.-L., Yang Q.-Q., Chen J.-R., Xiao W.-J. (2010). Organocatalytic asymmetric Sulfa-Michael/Michael addition reactions: A strategy for synthesis of highly substituted chromans with a quaternary stereocenter. Angew. Chem. Int. Ed..

[62] Hou W., Zheng B., Chen J., Peng Y. (2012). Asymmetric synthesis of polysubstituted 4-amino- and 3,4-diaminochromanes with a chiral multifunctional organocatalyst. Org. Lett..

[63] Wang X.-F., An J., Zhang X.-X., Tan F., Chen J.-R., Xiao W.-J. (2011). Catalytic asymmetric aza-Michael-Michael addition cascade: Enantioselective synthesis of polysubstituted 4-aminobenzopyrans. Org. Lett..

[64] Brandau S., Maerten E., Jørgensen K.A. (2006). Asymmetric synthesis of highly functionalized tetrahydrothiophenes by organocatalytic domino reactions. J. Am. Chem. Soc..

[65] Li H., Zu L., Xie H., Wang J., Jiang W., Wang W. (2007). Enantioselective organocatalytic double Michael addition reactions. Org. Lett..

[66] Ling J.B., Su Y., Zhu H.-L., Wang G.-Y., Xu P.-F. (2012). Hydrogen-bond-mediated cascade reaction involving chalcones: Facile synthesis of enantioenriched trisubstituted tetrahydrothiophenes. Org. Lett..

[67] Zu L., Xie H., Li H., Wang J., Jiang W., Wang W. (2007). Chiral Amine Thiourea-Promoted Enantioselective Domino Michael-Aldol Reactions between 2-Mercaptobenzaldehydes and Maleimides. Adv. Synth. Catal..

[68] Wang J., Xie H., Li H., Zu L., Wang W. (2008). A highly stereoselective hydrogen-bond-mediated Michael-Michael cascade process through dynamic kinetic resolution. Angew. Chem. Int. Ed..

[69] Yu C., Zhang Y., Song A., Ji Y., Wang W. (2011). Interplay of direct stereocontrol and dynamic kinetic resolution in a bifunctional amine thiourea catalyzed highly enantioselective cascade Michael-Michael reaction. Chem. Eur. J..

[70] Meninno S., Croce G., Lattanzi A. (2013). Asymmetric synthesis of trisubstituted tetrahydrothiophenes bearing a quaternary stereocenter via double Michael reaction involving dynamic kinetic resolution. Org. Lett..

[71] Breuer M., Ditrich K., Habicher T., Hauer B., Kebeler M., Stermer R., Zelinski T. (2004). Industrial methods for the production of optically active intermediates. Angew. Chem. Int. Ed..

[72] Patel R.N. (2000). Stereoselective Biocatalysis.

[73] Jarvo E.R., Miller S.J. (2002). Amino acids and peptides as asymmetric organocatalysts. Tetrahedron.

[74] Blaser H.U., Federsel H.J. (2011). Asymmetric Catalysis on Industrial Scale.

[75] Carter H.E. (1946). Azlactones. Org. React..

[76] Berkessel A., Cleemann F., Mukherjee S., Mueller T.N., Lex J. (2005). Highly efficient dynamic kinetic resolution of azlactones by urea-based bifunctional organocatalysts. Angew. Chem. Int. Ed..

[77] Berkessel A., Mukherjee S., Cleemann F., Mueller T.N., Lex J. (2005). Second-generation organocatalysts for the highly enantioselective dynamic kinetic resolution of azlactones. Chem. Commun..

[78] Jang H.B., Rho H.S., Oh J.S., Nam E.H., Park S.E., Bae H.Y., Song C.E. (2010). DOSY NMR for monitoring self-aggregation of bifunctional organocatalysts: Increasing enantioselectivity with decreasing catalyst concentration. Org. Biomol. Chem..

[79] Oh S.H., Rho H.S., Lee J.W., Lee J.E., Youk S.H., Chin J., Song C.E. (2008). A highly reactive and enantioselective bifunctional organocatalyst for the mechanolytic desymmetrization of cyclic anhydrides: Prevention of catalyst aggregation. Angew. Chem. Int. Ed..

[80] Rho H.S., Oh J.S., Lee J.W., Lee J.Y., Chin J., Song C.E. (2008). Bifunctional organocatalyst for methanolytic desymmetrization of cyclic anhydrides. Chem. Commun..

[81] Oh J.-S., Lee J.-W., Ryu T.H., Lee J.H., Song C.E. (2012). Self-assocation free bifunctional thiourea organocatalysts: Synthesis of chiral α-amino acids via dynamic kinetic resolution of racemic azlactiones. Org. Biomol. Chem..

[82] Kozlowski M.C., Morgan B.J., Linton E.C. (2009). Total synthesis of chiral biaryl natural products by asymmetric biaryl coupling. Chem. Soc. Rev..

[83] Noyori R., Takaya H. (1990). BINAP: An efficient chiral element for asymmetric catalysis. Acc. Chem. Res..

[84] Chen Y., Yekta S., Yudin A.K. (2003). Modified BINOL ligands in asymmetric catalysis. Chem. Rev..

[85] Terada M. (2008). Binaphthol-derived phosphoric acid as a versatile catalyst for enantioselective carbon-carbon bond forming reactions. Chem. Commun..

[86] Parmar D., Sugiono E., Raja S., Rueping M. (2014). Complete field guide to asymmetric BINOL-phosphate derived Bronsted acid and metal catalysis: History and classification by mode of activation; Bronsted acidity, hydrogen bonding, ion pairing, and metal phosphates. Chem. Rev..

[87] Hayashi T., Niizuma S., Kamikawa T., Suzuki N., Uozumi Y. (1995). Catalytic asymmetric synthesis of axially chiral biaryls by palladium-catalyzed enantioposition-selective cross-coupling. J. Am. Chem. Soc..

[88] Kakiuchi F., Le Gendre P., Yamada A., Ohtaki H., Murai S. (2000). Atropselective alkylation of biaryl compounds by means of transition metal-catalyzed C-H/olefin coupling. Tetrahedron Asymmetry.

[89] Bhat V., Wang S., Stoltz B.M., Virgil S.C. (2013). Asymmetric synthesis of QUINAP via Dynamic kinetic resolution. J. Am. Chem. Soc..

[90] Ros A., Estepa B., Ramírez-Lopez P., Álvarez E., Fernandez R., Lassaletta J.M. (2013). Dynamic kinetic cross-coupling strategy for the asymmetric synthesis of axially chiral heterobiaryls. J. Am. Chem. Soc..

[91] Hazra C.K., Dherbassy Q., Wencel-Delord J., Colobert F. (2014). Synthesis of axially chiral biaryls through sulfoxide-directed asymmetric mild C-H activation and dynamic kinetic resolution. Angew. Chem. Int. Ed..

[92] Zheng J., You S.-L. (2014). Construction of axial chirality by Rhodium-catalyzed asymmetric dehydrogenative Heck coupling of biaryl compounds with alkenes. Angew. Chem. Int. Ed..

[93] Gustafson J.L., Lim D., Miller S.J. (2010). Dynamic kinetic resolution of biaryl atropisomers via peptide-catalyzed asymmetric bromination. Science.

[94] Gustafson J.L., Lim D., Barrett K.T., Miller S.J. (2011). Synthesis of atropisomerically defined, highly substituted biaryl scaffolds through catalytic enantioselective bromination and regioseletive cross-coupling. Angew. Chem. Int. Ed..

[95] Mori K., Ichikawa Y., Kobayashi M., Shibata Y., Yamanaka M., Akiyama T. (2013). Enantioselevtive synthesis of multisubstituted biaryl skeleton by chiral phosphoric acid catalyzed desymmetrization/kinetic resolution sequence. J. Am. Chem. Soc..

[96] Shirakawa S., Wu X., Maruoka K. (2013). Kinetic resolution of axially chiral 2-amino-1,1′-biaryls by Phase-Transfer-Catalyzed N-Allylation. Angew. Chem. Int. Ed..

[97] De C.K., Pesciaioli F., List B. (2013). Catalytic asymmetric benzidine rearrangement. Angew. Chem., Int. Ed..

[98] Ma G., Deng J., Sibi M.P. (2014). Fluxionally chiral DMAP catalysts: Kinetic resolution of axially chiral biaryl compounds. Angew. Chem. Int. Ed..

[99] Yu C., Huang H., Li X., Zhang Y., Wang W. (2016). Dynamic kinetic resolution of biaryl lactones via a chiral bifunctional amine thiourea-catalyzed highly atropo-enantioselective transesterification. J. Am. Chem. Soc..

[100] Nicolaou K.C., Petasis N.A. (1984). Selenium in Natural Product Synthesis.

[101] Liotta D. (1987). Organoselenium Chemistry.

[102] Back T.G. (1999). Organoselenium Chemistry A Practical Approach.

[103] Wirth T. (2000). Topics in Current Chemistry: Organoselenium Chemistry.

[104] Petragnani N., Stefani H.A., Valduga C.J. (2001). Recent advances in selenocyclofunctionalization reactions. Tetrahedron.

[105] Ranganathan S., Muraleedharan K.M., Vaish N.K., Jayaraman N. (2004). Halo- and selenolactonisation: The two major strategies for cyclofunctionalisation. Tetrahedron.

[106] Wirth T. (2012). Organoselenium Chemistry: Synthesis and Reactions.

[107] Zhang H., Lin S., Jacobsen E.N. (2014). Enantioselective selenocyclization via dynamic kinetic resolution of seleniranium ions by hydrogen-bond donor catalysts. J. Am. Chem. Soc..

[108] Woong Lee J., Hi Ryu T., Suk Oh J., Yong Bae H., Bin Jang H., Eui Song C. (2009). Self-association-free dimeric cinchona alkaloid organocatalysts: Unprecedented catalytic activity, enantioselectivity and catalyst recyclability in dynamic kinetic resolution of racemic azlactones. Chem. Commun..

[109] Oh J.-S., Kim K.I., Song C.E. (2011). Enantioselective synthesis of α-deuterium labeled chiral α-amino acids via dynamic kinetic resolution of racemic azlactones. Org. Biomol. Chem..

[110] Afantitis A., Melagraki G., Sarimveis H., Koutentis P.A., Markopoulos J., Igglessi-Markopoulou O. (2006). A novel QSAR model for predicting induction of apoptosis by 4-aryl-4H-chromenes. Bioorg. Med. Chem..

[111] Conti C., Monaco L.P., Desideri N. (2011). Design, synthesis and in vitro evaluation of novel chroman-4-one chroman, and 2H-chromene derivatives as human rhinovirus capsid-binding inhibitors. Bioorg. Med. Chem..

[112] Kashiwada Y., Yamazaki K., Ikeshiro Y., Yamagishi T., Fujioka T., Mihashi K., Mizuki K., Cosentino L.M., Fowke K., Morris-Natschke S.L., Lee K.-H. (2001). Isolation of rhododaurichchromanic acid B and the anti-HIV principles rhododaurichromanic acid A and rhododaurichchromenic acid from *Rhododendron dauricum*. Tetrahedron.

[113] Zu L., Zhang S., Xie H., Wang W. (2009). Catalytic asymmetric oxa-Michael-Michael cascade for facile construction of chiral chromans via an aminal intermediate. Org. Lett..

[114] Yang W., Yang Y., Du D.-M. (2013). Squaramide-tertiary amine catalyzed asymmetric cascade Sulfa-Michael/Michael addition via dynamic kinetic resolution: Access to highly functionalized chromans with three contiguous stereocenters. Org. Lett..

